# President’s Address

**Published:** 1896-10

**Authors:** B. Fincké

**Affiliations:** Brooklyn, N. Y.


### PRESIDENT'S ADDRESS.

B. FINCKÉ, M. D., BROOKLYN, N. Y.

MEMBERS OF THE INTERNATIONAL HAHNEMANNIAN ASSOCIATION:—All the homœopathic world celebrates this year as the centennial of the discovery of Homœopathy through Samuel Hahnemann, when for the first time he stated the principle of healing under the formula, *Similia Similibus*.* Though from the oldest historical time up to his age it had been touched upon repeatedly, no physician had declared it with the lucidity which his inductive reasoning enabled him to enunciate it as a scientific principle of the first rank. He did not reason it out at his desk on theoretical grounds, but at once went to work practically to make the first experiment in 1790, by testing the medicine upon his own sound body. Only after six years of further research and practical experiments he arrived at the irrefutable principle that similars are cured by similars. *Similia Similibus Curantur*. This homœopathic mode of healing was *taught* by nobody before Hahnemann, and this is the main object of our celebration of the present centennial. There was, however, one point underlying his discovery without which he could not have succeeded. This was the necessity of using one remedy at a time in proving and healing. All his experiments would have amounted to nothing if he had employed mixtures for his investigations, as were commonly used by the physicians of his time. This, therefore, the *simplex*, was the *conditio sine*

---

* Versuch über ein neues Princip zur Auffindung der Heilkräfte der Arzneisubstanzen. *Hufeland's Journal*, II, 3d piece, 1796.

*qua non* by which he rose beyond the mass of his contemporaries, the recipes of which have, in consequence of his teachings, dwindled more and more, that even the old school at last has learned to some extent the precious lesson of administering only one remedy at a time. Hahnemann, of course, commenced the administration of his medicines in the form then in universal use. The dosology took the limit to be as much as the organism could stand. But it is known that Hahnemann always, even before his enunciation of the new principle, was moderate in his doses, and allowed them to act without repeating or interfering by other medicines as long as they acted well. Already a year after the publication of the new principle he declared "simplicity is the supreme law of the physician," and lauded Hippocrates in the words: "Only by this simplicity of his treatment in diseases he could see all that which he saw and at which we are astonished." We might therefore say that this law of simplicity, revived from Hippocrates, was the precursor of the law of healing which to-day we celebrate and without which the latter could not have been established. In the years following, Hahnemann continued his investigations at the hand of the law, and already in 1805 he pronounced the sentence that "In order to produce the most beneficial actions a single, simple remedy without any addition is always appropriate, if it is only the best selected, the most fitting in the right dose. It is *never* necessary to combine two of them." Here we have already the faint traces of his progress to the determination of the right dose and the invisible step to his momentous discovery —*Potentiation.* When the limits of posology hitherto had been the maximum which an organism in sickness was able to bear, his searching mind led him gradually to lessening his dose to a hitherto unheard-of degree, which startled the Nestor of the medical school of that age to the exclamation: "How can a 1-100,000 grain of Belladonna have any effect at all?" But Hahnemann had already given a millionth grain in disease, and he exclaims on this occasion, in 1801: "Will they understand at last how small, how infinitely small, the doses of the medicine must be in the sick state in order to affect the organism powerfully?" Such enormous progress had Hahnemann made in the few years following his discovery of the law of healing. Ameke remarks, justly: "No history reports, no writing shows us that ever a physician has searched with such ardor for the determination of dosology as we see in this, as the sharp-sighted, indefatigable, meditating Hahnemann." He used milk, sugar, alcohol, and water, as means of diminishing the hitherto accepted dose-quantities to render the medicine more appropriate to the susceptibility of the sick organism. This, also, is an invention of his own, for whoever before him used these inert substances as means of subdividing the crude materials into minute quantities according to his method? If, therefore, the half-homœopaths fall back upon the earlier period of Hahnemann's posology, they have to go back as far as 1797, when he cured a case of colicodynia with the powdered root of Veratrum-album in 4-grain doses, but only afterward his endeavor is known to diminish the doses, that already, in 1801, he speaks of infinitesimals, or infinitely small doses, and in 1805, when he announced the right dose as the fittest he could mean nothing else than its infinitesimality. So even before the appearance of *The Organon* for the first time in 1810, the foundation of potentiation was laid in this sentence, a fact which nobody can gainsay and which should be minded by the majority of our profession, which continues to adopt the practice of low potencies and low triturations exclusively, and have only a sneer at the high potencies which are now used more extensively than we know. They were the product of an evolution of posology which is the great and indisputable discovery of Hahnemann, one of the most important inventions which ever human genius has brought to light—potentiation (Ameke). We would therefore be amiss in our duty of celebrating the centennial of the Law of Similars if we should neglect the law next to the important law of remedy-selection, the law of Posology, viz., *Potentiation.* Well has Ameke said: "He found that by this mode of preparation (through diminution of substance) the adaptedness of many medicines, instead of decreasing, was directly unfolded, that in such a manner prepared remedial agents exerted an action which could not be obtained by crude substances. Furthermore, the surprising fact was revealed that medicinal substances could pass through so many degrees of preparation that neither physics nor chemics were able to detect in them any amount of matter and yet preserved a great power of healing: the most poisonous substances could in this manner be transformed into beneficial, never injurious, remedies, and easily decomposed substances, becoming inactive thereby, could be brought into a form in which they were no more exposed to decomposition, and remained or became more powerful instruments of healing in the hands of an educated physician."

When, sixteen years ago, the International Hahnemannian Association was founded, it was a matter of necessity, because Homœopathy was in danger of being destroyed at the hands of the large homœopathic body in our country for two reasons. One, the adherence to the low potencies and crude substances to the exclusion of the high potencies distinctly advocated and prescribed by Hahnemann, as witness in *The Organon* and *Chronic Diseases,* not to mention the writings in this respect besides. The other, pathological prescribing, because it was maintained that *Similia Similibus* did not cover the whole art and science of healing. To this came the preponderance of the surgical branch, which had very little use for potencies of any kind, and followed the lead of the enormous progress of modern surgery with its anæsthetics and disinfectants to the detriment of internal medicine, for they had little more need of internal medicine and used our vulnerary remedies in simple dilution of the tinctures for external application. There was, indeed, no hope for development of the Hahnemannian ideas with regard to potentiation. Therefore, the men who founded the International Hahnemannian Association did well to form a body of their own, which, in the course of time, adopted the motto: *Simplex simile minimum.* Not that the *Similia Similibus* was not sufficient to cover all these necessary elements of homœopathics, but to give an unequivocal expression of the aim of the Association that Homœopathy is not simply the application of a single simple remedy, whatever that may be, nor merely the similar one without regard to the dose in its simplicity, nor the infinitely small dose without regard to either, but that they all in close union form the complete formula of *Similia Similibus Curantur*. The remedy to be administered must be selected according to symptom-simility in a dose and preparation adapted to the susceptibility of the organism and similar to the life force. Whoever does not acknowledge these three necessary elements of the Hahnemannian principle, expressed by *Similia Similibus*, may be a very able physician or surgeon or gynæcologist, but he is not a Hahnemannian homœopathician (homœopathiker). We know very well that medicine moves in various directions, and nowhere the old adage is more true than here: *Practica est multiplex.* And there is freedom of opinion and action, and license enough for any mode of practicing according to or without principles which it is not necessary to further dilate upon. But this Association of ours, as it was founded and has worked with remarkable benefit for the cause of true Homœopathy and actual healing of the sick, does not rest upon the quicksand of fleeting opinion, but upon the inexorable, immutable law of healing expressed in our motto. Its outspoken purpose is to perfect the *Materia Medica Pura* by provings, repertories, and commentaries, to develop and bring to higher efficacy the posology of Hahnemann, which advocates high potencies from first to last, and to give physicians raised in the current routine posology of low potencies and crude substances the opportunity to study this subject by theory and practice. Any physician with reputable diploma is accepted through our institution of junior membership if he do not prefer to seek for immediate active membership. In this manner the Association offers to all the physicians who do not find in the existing bodies of learning the requisite instruction and information in regard to Hahnemannian posology an opportunity to become acquainted with it, and such searchers for truth are always welcome as co-operators in our good cause.

We know very well that low potencies also cure, and that we obtain valuable provings from them as far as it goes; but experience has taught us that high potencies will do the same, and, as a general rule, accomplish more. Our antagonists cannot be brought by any means to test the matter to the bottom, as the members of this Association have done. They are satisfied with the results of their therapeutic measures, and find no need of seeking for something better, especially at the expense of so much time, labor, and money as the preparation of high potencies requires. They consider Hahnemann to have been in his dotage when he taught potentiation as the fundamental principle of posology, but ignore his declaration that as early as 1801 he blamed the short-sightedness of his cotemporaries, who could not understand "how small, how infinitely small, the dose of the remedies in the sick state have to be in order to strongly affect the body." To urge the necessity of using low potencies because Hahnemann, in his earlier homœopathic years, had applied them, is therefore unfair, and not to be countenanced; hence the potencies up to the twelfth centesimal, which is set up by them as a limit to potentiation, are not infinitely small, but only comparatively small—*i. e.*, when compared with the doses used by the allopathic school. In talking about this matter we do by no means wish to dictate, but at the same time we wish to oppose dictation on the other side, which has no foundation at all in the pretended teaching and practice of Hahnemann. We know very well that it is human nature if something unusual is proposed which at first seems incompatible with the understanding, that the opposition is roused with reference to the merits of the subject. It is the immediate resistance to the shock received which, however, furnishes only the proof that it is received. It depends upon the calibre of the recipient whether the resistance is continued merely to ward off the shock which continues with it, or whether the resistance dies out with the reception. But if the shock continues it enters into the understanding, producing a new motion in a direction different from the one hitherto followed. This is natural and cannot be helped. May they who cannot receive the excellent and beneficent ideas regarding potentiation first promulgated by Hahnemann, resist persistently to their entrance into their understanding and follow their own chosen direction, but they must not wonder at the repeated shocks which they will have to bear upon their ignoring indifference and their opposition to the further development of Hahnemann's teaching. Those opponents have to rue it sooner or later, because the march of science is ever forward, not backward. It may for a while be diverted into a blind alley, but invariably it will turn back into the main road of increasing knowledge. Such a blind alley is the limit set to potentiation upon grounds which are taken from a deduction of investigations in departments not immediately concerning Homœopathy, avoiding the induction from experiments made in order to prove the action of highly potentiated medicine. If the great mathematicians calculate the limit of substances and determine the size of their atoms, it is nothing but a repetition of the inductive Hahnemannian method which demands the experience, experiment, and correct observation of a trained philosopher. It is not philosophical, however, to apply the undoubtedly correct computations of those mathematicians without further thought to the high potencies of Homœopathy because the application is faulty in its premise, that the high potencies owe their action to the matter from which they are derived. It is plainly a begging the question and an evasion, because the point to be proved by experiment is asserted by reference to mathematical computation of matter. The proof of a thing cannot be furnished by a mere psychical process in our mind which has no reality outside of it. The thing can only be proved by the observation of its action upon other things. Matter is a thing. Force is a thing. Force acts through matter and matter through force as far as it goes. For it does not go into infinity, as those mathematicians themselves prove who arrive by their calculations at the ultimate atom of matter. We can and will not contest their results, they spring from their eminent ability to follow their legitimate search into the last recesses of matter, and find it in a conceived computed minuteness of the atom. But we claim that high potencies have nothing in common with matter. The substances from which they are derived hold the specific force which we need for action upon the living body in suspense; they hold the forces as a vessel holds water, and by proper manipulation invented and first taught and introduced by Hahnemann, they are liberated from their prison and spread all over the inert materials used for this liberation and expansion, which surpasses all conception of minuteness of quantity and reaches far over the calculated atoms of the physical and mathematical genius. The postulate, that we have to prove the laws of physics to be incorrect in order to prove our potentiation to be correct, does not meet the question and evades the point at issue. For we do not pretend to handle medicinal forces without matter, since we can carry them up by unmedicinal inert solids and fluids. Nothing is truer in the world, at least accessible to us, than that no force can exist without matter. But the *rôle* assigned to matter by the materialistic philosophers is a false one, viz., that force emanates from or is created by matter and dispenses with the highest conception which man is able to conceive that there is an Almighty Creator of the world and of all its things which He preserves according to certain laws, the most important of which have been found and proclaimed by illustrious men from age to age. We must never forget that science always lags behind the creation, and serves merely as the receptacle or vehicle for the human race to carry on the conceptions gleaned more and more from the acquired knowledge of the universe. The milliards of years calculated and required to account for the evolution of the present state of things may satisfy the pride of certain minds for their ability to penetrate time and space into the farthest distances. But after all, it is but calculation based upon premises which can by no means be as certain as they pretend them to be. They are idle speculations hiding behind computations and conclusions diverting the mind from the more serious problems which are yet to be solved by scientific labor. Certain it is that the world *was* before any science in our sense was thought of. The laws have been active in their supervising work before any man eliminated them from a multitude of facts. The creation goes on all the same as ever, and its mystery is as great as ever to the searching mind. The multitude of systems of suns increases with the greater perfection of our instruments, and there is no end to our inquiries, which, satisfied in one point, raise numerous others demanding investigation; and so it goes on year for year, and the wisest men, having given their best services for their fellow-men, disappear after a while and give place to a succession of others, who, in the favorable case, will take up their labors where they left them, if they are finding it worth while, in order to go the way of all flesh again when their work is done. Thus the heritage left to us by our great and beloved master is a sacred obligation come down to us for a period of a hundred years. What a variety of phenomena does not the history of medicine present during that time! Hahnemann commenced his clearing in the primeval forest of medicine with such means as he had, and his time furnished him in the steady continuation of his work whilst he was cutting down the giants of the thousand-year old trees of prejudice, burning up the brush of opposition to his teachings, ploughing the ground of investigation and sowing the seeds of his experience in it; whilst he destroyed the wild beasts of envy and slander with the weapons of his intellect; whilst he drained the swamps of routine and irrigated the barren soil of disease with the nourishing water of appropriate remedies, whilst he built his home of a logical system of homœopathics for the ready acceptance of his benevolent hospitality by those needing protection from the attacks of inimical influences abroad. Whilst he was busy doing all this the wilderness around him continued in its aboriginal force as the abode of the children of the soil which, before Hahnemann, they occupied as their hunting-ground, and now they considered him as an intruder, who interfered with their time-honored customs and traditions. These wild Indians of science went for his scalp many a time, but in vain, for the Great Spirit protected him and his work. Instead of going to work themselves to clear the forest and place the soil under cultivation for nourishing them decently and habituating their race to peace and leading them on to civilization, they carried the warfare repeatedly into the precincts of a man whom they hated with the hatred of a fiend. But they could not succeed, because the work of this man and of his successors was under the protection of the Most High. The clearing increased in extent and usefulness. The home built in the beginning has been multiplied, and many and larger buildings have been erected in the course of time for the instruction of pupils and for the healing of the sick; and apostles go out to teach and do as Hahnemann did, and break the ground for the new gospel of medicine first revealed by him everywhere on the globe.

[TO BE CONTINUED.]

